# Country-level incidence of Alzheimer disease and related dementias is associated with increased omega-6-PUFA consumption

**DOI:** 10.1038/s43856-025-01059-3

**Published:** 2025-07-31

**Authors:** Timothy H. Ciesielski, Giuseppe Tosto, Razaq O. Durodoye, Farid Rajabli, Rufus O. Akinyemi, Goldie S. Byrd, William S. Bush, Brian W. Kunkle, Christiane Reitz, Jeffery M. Vance, Margaret A. Pericak-Vance, Jonathan L. Haines, Scott M. Williams

**Affiliations:** 1 Department of Population and Quantitative Health Sciences, Case Western Reserve University School of Medicine, Cleveland, OH USA; 2 Taub Institute for Research on Alzheimer Disease and the Aging Brain, Department of Neurology, Columbia University College of Physicians and Surgeons, New York, NY USA; 3 John P. Hussman Institute for Human Genomics, University of Miami Miller School of Medicine, Miami, FL USA; 4 Neuroscience and Ageing Research Unit, Institute for Advanced Medical Research and Training, College of Medicine, University of Ibadan, Ibadan, Oyo State Nigeria; 5 Maya Angelou Center for Health Equity, Wake Forest University School of Medicine, Winston-Salem, NC USA; 6 Cleveland Institute for Computational Biology, Case Western Reserve University, Cleveland, OH USA

**Keywords:** Alzheimer's disease, Epidemiology

## Abstract

**Background:**

Clinical and genetic studies have implicated lipid dysfunction in Alzheimer Disease (AD) pathogenesis. While the etiologic impact of lipid intake on individuals is receiving attention, the role of food systems in shaping community-level incidence remains uncharacterized.

**Methods:**

Mean country-level lipid intakes were compared to Age-Standardized Alzheimer-and-other-Dementia Incidence Rates (ASAIR) in 183 countries across all inhabited continents. Free-knot penalized spline regression and multivariable-adjusted linear regression, including a lag between intake and incidence, were used to assess the relationships between five lipid intakes and ASAIR. Validation was conducted using longitudinal within-country changes between 1990 and 2019.

**Results:**

Here we show that omega-6 Polyunsaturated-Fatty-Acid (omega-6) intake exhibits a positive linear relationship with ASAIR (multivariable-adjusted model: *β* = 2.44; 95%CI: 1.70, 3.19; *p* = 1.38 × 10^−9^). ASAIR also increases with saturated-fat, trans-fat, and dietary-cholesterol up to a threshold. The association between omega6-PUFA and ASAIR is confirmed using longitudinal intake changes. The scale of predicted benefits varies by country but, our results predict a 2 standard deviation decrease (−3.8% as a percent of daily energy intake) in omega-6 intake would reduce ASAIR by 8% in the US. This level of consumption has already been achieved in 20 countries. If our other findings are validated in future work, decreasing all four lipids could potentially yield large ASAIR reductions (in the US: a 35% decrease).

**Conclusions:**

Higher levels of omega-6 consumption associate with increased ASAIR. Thus, decreasing omega-6 consumption on the country-level may have substantial benefits in reducing the burden of dementia.

## Plain language summary

Scientific evidence from several fields indicates that lipids (fats) play a role in Alzheimer Disease development. However, studies conducted on the individual-level are not well suited to inform population-level interventions. In other words, we need population-based approaches to study how fat intakes vary between people in different countries and food systems. Here we used statistical models to adjust for potential biases, and we observed that the incidence of Alzheimer Disease and other dementias increased with mean country-level intake of omega-6 polyunsaturated fatty acids (omega-6). It also increased with saturated-fat, trans-fat, and cholesterol intake up to a threshold. The country-level omega-6 finding was validated in a second set of analyses that evaluated changes in fat intake over time. If future research confirms these observations, they indicate that incidence of dementia could be reduced by lowering the mean intake of omega-6 globally.

## Abstract (web summary)

Ciesielski et al. address the weaknesses of individual-level studies for population-level inference on the role of lipid consumption in dementia. In cross-sectional and longitudinal analyses of 183 countries, they find that the age-standardized incidence of dementia increases with mean country-level intake of omega6 polyunsaturated fatty acids.

## Introduction

Convergent evidence from genetics, neuropathology, laboratory experiments, and epidemiology indicates that lipids play a central role in the development of Alzheimer Disease (AD)^1–9^. However, the relative importance of different lipids remains unclear, and systematic review of individual-level epidemiologic studies have yielded equivocal results^10^. This is not surprising given the intrinsic difficulties in this field. Large cohort studies rarely have precise dietary information on specific lipid intakes over decades and, and even where these data are available, the lipid intakes among individuals do not often vary enough within a study population to discern patterns of association. Additionally, multiple categories of lipids are not usually available in one dataset, so their relative associations often cannot be compared in one model. Furthermore, the potential for nonlinear relationships is rarely evaluated, and the difference between intake and circulating levels is not often explicitly considered^11^. Having said this, well-designed prospective studies on the individual level should eventually resolve many issues regarding the most appropriate levels of intake of lipids for AD prevention. However, this will not provide us with optimal answers on the population level or low agency intervention level^12,13^.

If we want to learn about what is best for our food systems and policies then we have to acknowledge that the individual level studies we use for individual level intervention may not be optimal when considering population level phenomena^12^. For this related but distinct task we need to consider the appropriate unit of analysis^13^. Fortunately, we now have data that can enable cross-sectional and longitudinal analyses on the country level. Mean intake levels for several lipid categories have now been measured concurrently across countries and they vary substantially among them^14,15^. The range and density of these data should allow for: 1) resolution of associations with AD incidence, and 2) the use of free-knot penalized spline regression^16^ to assess for nonlinear relationships. The ecological and individual level analytic approaches both depend on physiologic needs but they are distinct and complementary. The country level approach is not designed to tell people how much lipid to eat to avoid disease, and the individual level studies are not designed to set targets for food delivery systems that lower disease incidence.

Here we obtained country-level data on five lipid intakes^14,15^, and compared them to age-standardized Alzheimer Disease Incidence rates (ASAIR)^17^ using free-knot penalized spline regression and linear regression. We then attempted to validate our findings using longitudinal analyses that compared changes in country-level lipid intakes over 30 years to changes in ASAIR. These analyses do not seek to make inferences about individual level intakes and the risk of AD for individuals. Thus, the ecological fallacy is not a relevant concern as both the exposure and the outcome are on the country-level. Country-level dietary data is the most appropriate data for studying country-level incidence rates and country-level interventions. In this study we show that omega-6 polyunsaturated-fatty-acid (omega-6) intake exhibits a positive linear relationship with ASAIR, and that ASAIR also increases with saturated-fat, trans-fat, and dietary-cholesterol up to a threshold. Importantly, longitudinal intake changes confirm the association between omega-6 and ASAIR. This corroboration indicates that lowering the mean intake of omega-6 could reduce ASAIR in many countries.

## Methods

Previously published reports were used to obtain ASAIR^17^ and mean intake for 5 lipids^14,15^ across 184 countries. The lipids were: omega-6, long chain omega-3 polyunsaturated fatty acids (omega-3), saturated fat, trans-fat, and dietary cholesterol. One country, the Maldives, was eliminated from the analyses as it was an extreme outlier with respect to omega-3 (>9 standard deviations above the global mean). The lipid estimates were produced by the Nutrition and Chronic Diseases Expert Group (NutriCoDE) for the 2010 Global Burden of Diseases (GBD), Injuries, and Risk Factors Study^14,15,18^. They were intended to estimate the mean country-level intake among male and female adults over 20 years of age. Details have been previously published^14^, but in brief, the authors used PubMed to identify national survey data, and then identified alternative sources for countries with no national survey data. These alternative sources included: the WHO stepwise approach to Surveillance Database, the WHO Global Infobase, as well as large epidemiologic cohorts and other household surveys. A total of 266 distinct data sources were identified and this list included food frequency questionnaires, single and multiple response dietary recalls, as well as household budget data. The authors then used a Bayesian hierarchical imputation model to harmonize the estimates from these diverse sources^14,15,18^.

**Fig. 1:**
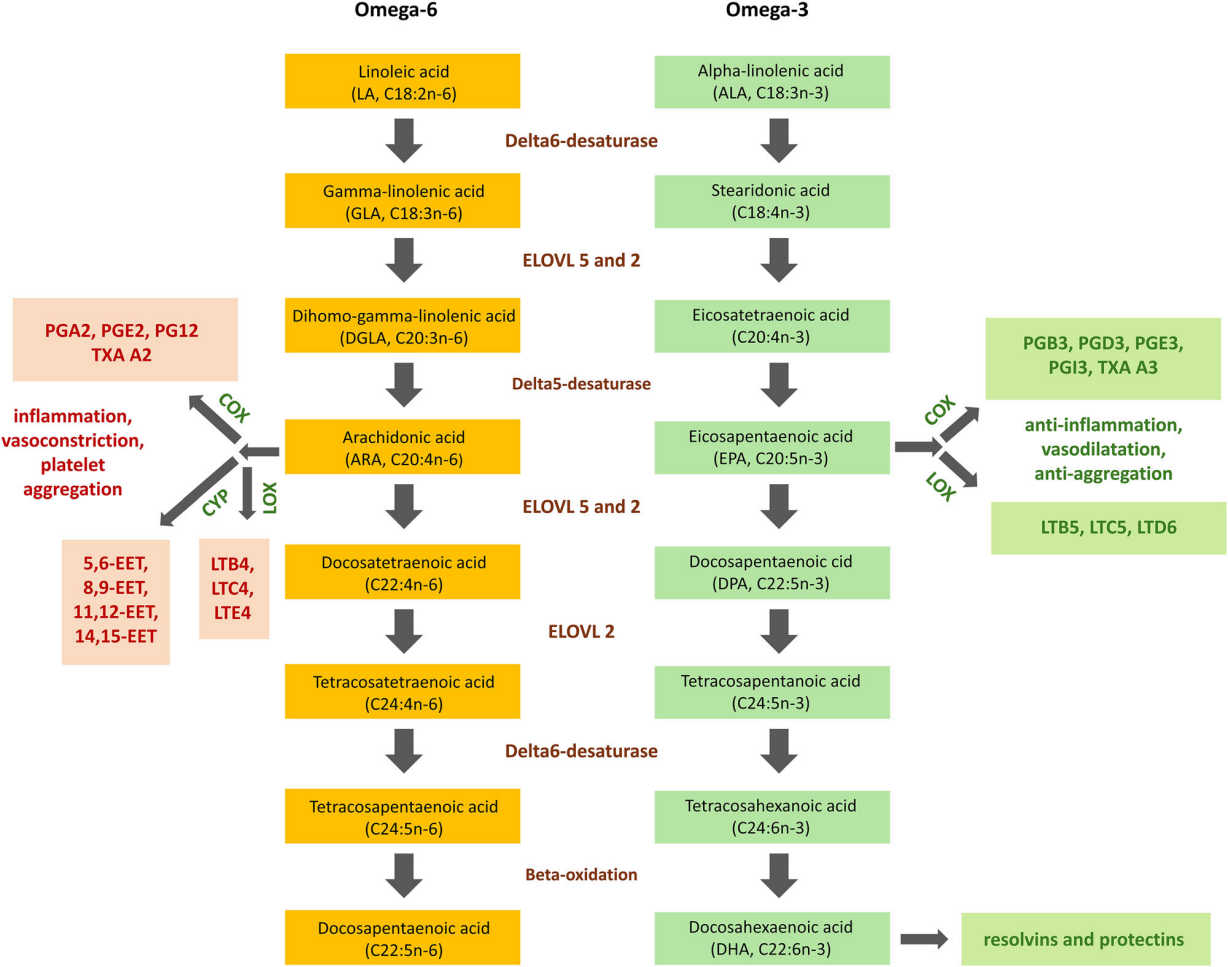
The shared biochemical pathway for omega-6 and omega-3 processing. This lipid processing pathway is central to research in omega-3 and omega-6. The same set of enzymes process both of these lipid classes, and thus an excess of omega-6 can alter and impair omega-3 processing. In short, an imbalance in substrates can produce a corresponding imbalance in products. The omega-6 products are largely proinflammatory mediators, while the omega-3 products are primarily inflammation resolving factors. This biochemistry predicts that excess omega-6 will drive excess inflammation. This figure is adapted from ref. ^52^ (reuse under the Creative Commons Attribution license; http://creativecommons.org/licenses/by/4.0/).

Omega-3 intake was estimated in 2 subgroups: Plant-based (primarily alpha linolenic acid [ALA]) and seafood based (primarily eicosapentaenoic acid [EPA], docosahexaenoic acid [DHA] and the intermediates listed on the bottom half of Fig. 1). Because ALA can be endogenously converted to EPA and DHA (long chain omega-3; Fig. 1)^19–22^, this conversion is needed to estimate the long chain omega-3 that a given diet provides. Failing to account for this biochemistry would yield underestimates, and even with low conversion rates, this bias could be large for countries that have low seafood and high plant-based omega-3 intake^19–22^. Thus, our long-chain omega-3 estimates account for this endogenous conversion with the previously described calculations^19^. We used a sex-averaged mean ALA to EPA conversion rate of 15%, acknowledging that a variety of genetic, environmental, and intrinsic biological influences on the conversion rate are not available on the country level. The omega-6 data were not subdivided by Micha et al.^14,15^ and thus represent intake of the full set of omega-6 species (Fig. 1).

ASAIR estimates were produced by the GBD Disease and Injury Incidence and Prevalence Collaborators and downloaded from the IHME Global Health Data Exchange (http://ghdx.healthdata.org/gbd-results-tool) on March 20, 2020^17^ Incidence rates in this study are for AD and other dementias (national autopsy programs are not available for diagnostic confirmation at scale). The authors stated that dementia was rarely identified before 40 years of age, so they excluded these cases^17^. Age standardization was conducted to account for country-level differences in population age distributions and age of dementia onset. The authors used the GBD world population age standard for this process, and this approach is described in appendix 1 of Vos et al., 2020^23^. In short, the mean of the age-specific proportional distributions for each nation were estimated using the UN Population Division World Population Prospects data. These were used to create a standard population age structure to facilitate cross national comparisons that control for population age structure (details available in section 3.3.4, on page 56 of appendix 1 from Vos et al.)^23^. Lipid intake data was from 2010 and ASAIR values were from 2019. This 9-year time-lag aligns with optimal lags estimated for environmental factors in AD epidemiology^24^. This analysis used only country-level data that are publicly available from previously published papers and as such was not human subject research.

### Statistics and reproducibility

#### Cross-sectional analyses with a lag

To assess the shape of the 5 lipid-ASAIR relationships, we implemented free-knot penalized regression splines using the MGCV package in R (Mixed GAM Computation Vehicle with Automatic Smoothness Estimation)^16^. The three lipid intakes that demonstrated non-linear relationships with ASAIR in these bivariate regression spline models were specified in the subsequent multivariable-adjusted model with spline terms. Limited covariate data were available for confounding adjustments, but this model was adjusted for all other lipids and country development level based on per capita Gross National Income (GNI)^25^. The per capita GNI for each country in 2010 was calculated using the World Bank Atlas method as described in Blencowe et al., 2012^25^ (0 = Low-income economies, 1 = Lower-middle-income, 2 = Upper-middle-income and 3 = High-income economies). Since the three nonlinear relationships appeared to be linear on either side of a threshold in the bivariate spline models, we also used the thresholds to create 2 strata for each of these lipids (strata within which the lipid-ASAIR relationship appears linear). We then specified linear terms for these lipids and reran the multivariable-adjusted regression model in the two strata separately (to obtain βs for the linear regions of the lipid-ASAIR relationship).

#### Longitudinal analyses

Finally, we compared the percent change in lipid intakes from 1990 to 2010^14,15^ to the percent change in ASAIR between 1990 and 2019^17^ with linear regression. We first assessed unadjusted linear regression models within the linear regions of the relationship identified in the analyses described above. Any lipid that yielded significant findings was then evaluated in a multivariable-adjusted model that was adjusted for all other lipids. To identify distinct associations on opposite sides of the thresholds for saturated fat, trans-fat, and dietary cholesterol, we tested for interaction with a dichotomous dummy variable for change-occurred-above-or-below threshold. Finally, we adjusted for changes in country development level based on per capita GNI. This variable was coded as the development level change from 1990 to 2010. Development levels were not available for 27 countries in 1990, but we were able to obtain substitute baseline values for 24 of these countries by using development estimates from 1991 or 1992^26^. Thus, only Montenegro, Serbia, and Timor-Leste were excluded due to missing baseline development data in any of these years. Note that the lipid intake information is the percent change in the country-level intake between 1990 and 2010, and the incidence data is percent change in the annual age standardized incidence between 1990 and 2019. Since these units differ from the prior analyses, these data can be used to corroborate association directions, but the association magnitudes are not directly comparable. Descriptive statistics, scatterplots, and spearman rank correlations were obtained using SAS 9.4 (Cary, NC); all other analyses were conducted with R 4.2.1. Analyses were conducted using standard commands for these programs. No one-sided tests were used. For a list of countries, as well as their ASAIR and lipid intake estimates see Supplementary Data File 1. The raw data is available in Supplement Table 1 from Li et al., 2022 (download available at https://www.frontiersin.org/journals/aging-neuroscience/articles/10.3389/fnagi.2022.937486/full^17^) and eTable 3 and 4 from BMJ 2015;350:h1702 (download available at https://www.bmj.com/content/350/bmj.h1702/related^15^).

### Reporting summary

Further information on research design is available in the Nature Portfolio Reporting Summary linked to this article.

## Results

The mean ASAIR among the 183 countries was 91.6 new cases per 100,000 per year with a standard deviation of 11.7 (Table 1). Mean country-level omega-6, saturated fat, and trans-fat intakes were 5.1%, 11.6%, and 1.1% of total energy intake, respectively, and the mean intakes for cholesterol and omega-3 were 323 and 249 mg/day, respectively. Scatterplots indicated positive relationships between 4 of the lipids (omega-6, saturated fat, trans-fat, and dietary cholesterol) and ASAIR (Supplementary Figs. 1–5; Supplementary Information). Spearman rank correlations revealed that the strongest magnitude and most significant bivariate relationships were: 1) dietary cholesterol and GNI, and 2) omega-6 and ASAIR (Table 2). GNI was positively correlated with ASAIR and all of the lipids except for saturated fat (Tables 2 and 3).

**Table 1:** Descriptive statistics for the 183 countries

	Mean	Standard deviation	Median	25th percentile	75th percentile	Min	Max
Outcome Variable							
Age Standardized Alzheimer Disease Incidence (ASAIR)^a^ (new cases annually per 100,000)	91.6	11.7	90.0	80.0	100.0	60.0	110.0
Exposure Variables							
Omega6-Polyunsaturated Fatty Acid (omega-6)^b^ (% of total daily energy intake)	5.1	1.9	4.9	3.7	6.3	1.2	12.5
Long Chain Omega-3 Polyunsaturated Fatty Acid (omega-3)^b^ (mg/day)	323	273	261	153	403	35	2011
Saturated Fat^b^ (% of total daily energy intake)	11.6	4.5	11.0	8.8	13.7	2.3	27.5
Trans-Fat^b^ (% of total daily energy intake)	1.1	0.7	1.0	0.8	1.2	0.2	6.5
Dietary Cholesterol^b^ (mg/day)	249	55	247	209	286	97	439
Gross National Income^c^	1.6	1.1	2	1	3	0	3

^a^ASAIR estimates were obtained from Li et al., 2022^17^. They were produced by the *GBD Disease and Injury Incidence and Prevalence Collaborators* and downloaded from the IHME Global Health Data Exchange (http://ghdx.healthdata.org/gbd-results-tool) on March 20, 2020.

^b^Lipid estimates were obtained from Micha et al., 2015^14,15,18^. They were produced by the *Nutrition and Chronic Diseases Expert Group* (NutriCoDE) for the 2010 Global Burden of Diseases, Injuries, and Risk Factors Study, and they used a Bayesian hierarchical imputation model harmonize these estimates from diverse source data^14,15,18^.

^c^The development level (based on per capita GNI) for each country in 2010 was calculated using the World Bank Atlas method as described in Blencowe et al., 2012^25^ (0 = Low-income economies, 1 = Lower-middle-income, 2 = Upper-middle-income and 3 = High-income economies).

**Table 2:** Spearman rank correlations between variables: bivariate analyses with *p*-values in parentheses

	Omega6-^a^ (% of total daily energy intake)	Omega-3^a^ (mg/day)	Saturated Fat^a^ (% of total daily energy intake)	Trans-Fat^a^ (% of total daily energy intake)	Dietary Cholesterol^a^ (mg/day)	Gross National Income^c^
Omega-3^a^ (mg/day)	0.007 (9.28 × 10^−1^)					
Saturated Fat^a^ (% of total daily energy intake)	−0.207 (4.83 × 10^−3^)	0.357 (6.95 × 10^−7^)				
Trans-Fat^a^ (% of total daily energy intake)	0.237 (1.23 × 10^−3^)	0.063 (3.99 × 10^−1^)	0.078 (2.91 × 10^−1^)			
Dietary Cholesterol^a^ (mg/day)	0.323 (8.02 × 10^−6^)	0.370 (2.64 × 10^−7^)	0.091 (2.22 × 10^−1^)	0.176 (1.72 × 10^−2^)		
Gross National Income^b^	0.323 (8.45 × 10^−6^)	0.435 (7.35 × 10^−10^)	0.047 (5.29 × 10^−1^)	0.353 (9.82 × 10^−7^)	0.634 (6.21 × 10^−22^)	
ASAIR^c^ (new cases annually per 100,000)	0.514 (9.66 × 10^−14^)	0.059 (4.28 × 10^−1^)	0.144 (5.12 × 10^−2^)	0.356 (7.41 × 10^−7^)	0.371 (2.30 × 10^−7^)	0.332 (4.39 × 10^−6^)

^a^Lipid estimates were obtained from Micha et al., 2015^14,15,18^. They were produced by the *Nutrition and Chronic Diseases Expert Group* (NutriCoDE) for the 2010 Global Burden of Diseases, Injuries, and Risk Factors Study, and they used a Bayesian hierarchical imputation model harmonize these estimates from diverse source data^14,15,18^.

^b^The development level (based on per capita GNI) for each country in 2010 was calculated using the World Bank Atlas method as described in Blencowe et al., 2012^25^ (0 = Low-income economies, 1 = Lower-middle-income, 2 = Upper-middle-income, and 3 = High-income economies).

^c^ASAIR estimates were obtained from Li et al., 2022^17^. They were produced by the *GBD Disease and Injury Incidence and Prevalence Collaborators* and downloaded from the IHME Global Health Data Exchange (http://ghdx.healthdata.org/gbd-results-tool) on March 20, 2020 (*n* = 183 countries).

**Table 3:** Distributions of lipids and ASAIR by Gross National Income Level

Variable	Gross National Income^c^	Mean	Standard Deviation	Median	25th Percentile	75th Percentile	Min	Max	*N*
Omega6^b^ (% of total daily energy intake)	0	4.5	1.5	4.3	3.3	5.3	2.2	8.5	35
	1	4.1	1.7	3.9	3.0	5.2	1.2	8.3	53
	2	6.3	2.0	6.2	4.7	7.5	2.8	12.5	49
	3	5.4	1.6	5.3	4.2	6.3	2.7	9.9	46
Omega-3^b^ (mg/day)	0	186	152	130	99	197	50	712	35
	1	276	192	200	114	416	35	793	53
	2	343	259	268	190	414	58	1387	49
	3	462	363	321	269	530	156	2011	46
Saturated Fat^b^ (% of total daily energy intake)	0	10.4	3.4	10.9	8.7	13.0	2.3	16.7	35
	1	13.2	6.4	12.0	9.1	15.6	3.2	27.5	53
	2	10.7	3.6	9.4	8.3	11.8	6.2	21.4	49
	3	11.8	2.3	11.9	10.2	13.5	6.9	16.9	46
Trans-Fat^b^ (% of total daily energy intake)	0	0.9	0.4	0.8	0.7	0.8	0.4	2.5	35
	1	1.2	1.1	1.0	0.8	1.1	0.5	6.5	53
	2	1.1	0.5	1.0	0.9	1.2	0.5	3.6	49
	3	1.3	0.7	1.1	1.0	1.4	0.2	4.0	46
Dietary Cholesterol^b^ (mg/day)	0	190	32	189	175	206	97	282	35
	1	236	50	232	202	263	121	347	53
	2	274	49	262	243	289	194	439	49
	3	282	35	285	252	307	215	348	46
ASAIR^a^ (new cases annually per 100,000)	0	86	9	80	80	90	70	110	35
	1	89	12	90	80	100	60	110	53
	2	94	11	100	80	100	80	110	49
	3	96	11	90	90	110	80	110	46

^a^ASAIR estimates were obtained from ^Li et al., 2022,17^. They were produced by the *GBD Disease and Injury Incidence and Prevalence Collaborators* and downloaded from the IHME Global Health Data Exchange (http://ghdx.healthdata.org/gbd-results-tool) on March 20, 2020 (n = 183 countries).

^b^Lipid estimates were obtained from Micha et al., 2015^14,15,18^. They were produced by the *Nutrition and Chronic Diseases Expert Group* (NutriCoDE) for the 2010 Global Burden of Diseases, Injuries, and Risk Factors Study, and they used a Bayesian hierarchical imputation model harmonize these estimates from diverse source data^14,15,18^.

^c^The development level (based on per capita GNI) for each country in 2010 was calculated using the World Bank Atlas method as described in Blencowe et al., 2012^25^ (0 = Low-income economies, 1 = Lower-middle-income, 2 = Upper-middle-income, and 3 = High-income economies).

### Cross-sectional analyses with a lag

**Fig. 2:**
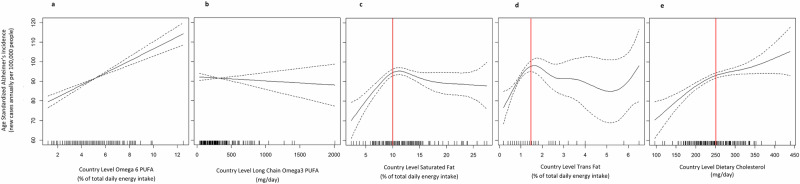
Free-knot penalized splines showing the relationship between country-level lipid intakes and ASAIR. Incidence is on the y-axis and the y axis range is the same for all 5 plots, for ease of comparison. Each plot depicts the penalized regression spline for a distinct class of country-level lipid data: **a** omega-6, **b** omega-3, **c** saturated fat, **d** trans-fat, and **e** dietary cholesterol intake. The dashed lines depict 95% CIs and the hash marks along the x-axis depict data density (*n* = 183 countries). Estimates are imprecise in regions of low data density. Nonlinear relationships were detected by the generalized cross-validation (GCV) process for saturated fat, trans-fat, and dietary cholesterol (**c**–**e**). Apparent thresholds are indicated with a vertical red line.

Bivariate (unadjusted) free-knot penalized spline regression analyses revealed a linear relationship between country-level ASAIR and both omega-6 and omega-3 (Fig. 2: a and b), but non-linearity was detected for the remaining three lipids: saturated fat, trans-fat, and cholesterol intake (Fig. 2c–e). ASAIR increased with saturated fat up to ~10% of total energy intake and with trans-fat up to ~1.5% total energy intake. ASAIR also increased with dietary cholesterol intake up to ~250 mg/day (approximate inflection point in the spline). Below these thresholds, the relationships appeared linear, while above these thresholds data was sparse. Thus, the slopes in the high intake regions of the exposure distribution could not be estimated with confidence, although the plots generally showed slopes near zero (Fig. 2).

A multivariable linear regression model adjusted for all other lipids and country development level (per capita GNI), revealed a positive association between omega-6 and ASAIR (Table 4; *β* = 2.44; 95%CI: 1.70, 3.19; *p* = 1.38 × 10^−9^). For each 1 unit increase in omega-6 (a 1% increase - as a percent of total energy intake) ASAIR increased by 2.44 cases per 100,000 people per year. For omega-3 there was a subtle negative trend, but the relationship was not significant (*β* = −0.004; 95%CI: −0.010, 0.001; *p* = 1.11 × 10^−1^). Because the remaining lipids were modeled with nonlinear splines, it was not appropriate to estimate a single slope across the full exposure distribution for these lipids. However, as noted above, the remaining lipid-ASAIR relationships appeared linear below lipid-specific thresholds. Thus, for the three remaining lipids, we stratified by that lipid’s specific threshold and reran the multivariable model using a linear term for that lipid. This allowed us to estimate slopes (βs) within the linear regions of the relationship. Below the threshold of 10% of total energy intake, we found a significant association between saturated fat and ASAIR (β = 2.67; 95%CI: 1.23, 4.11; *p* = 5.71 × 10^-4^). This relationship was not evident above the threshold (*β* = 0.04; 95%CI: −0.44, 0.53; *p* = 8.62 × 10^−1^). Similarly, trans-fat intake was significantly associated with ASAIR below the threshold of 1.5% total energy (*β* = 7.35; 95%CI: 0.79, 13.90; *p* = 2.96 × 10^−2^), but not above the threshold (*β* = 0.70; 95%CI: −1.89, 3.29; *p* = 6.03 × 10^−1^). Finally, the relationship between cholesterol intake and ASAIR was significant below the threshold of 250 mg/day (*β* = 0.15; 95%CI: 0.07, 0.23; *p* = 5.98 × 10^−4^), but not above it (*β* = 0.02; 95%CI: −0.03, 0.07; *p* = 3.61 × 10^−1^).

**Table 4:** Results from regression models of ASAIR on the country-level

	Change in ASAIR associated with a 1 unit increase in country-level lipid (β)	95% CI	*p* value
Omega-6 (% of energy intake) ^a^	2.44	1.70, 3.19	1.38 × 10^−9^
Omega-3 (mg/day) ^a^	-0.004	−0.010, 0.001	1.11 × 10^−1^
Gross National Income ^a^	0.29	−1.341.92	7.28 × 10^−1^
Saturated Fat (% of energy intake) ^b^			
Below the threshold of 10	2.67	1.23, 4.11	5.71 × 10^−4^
Above the threshold of 10	0.04	−0.44, 0.53	8.62 × 10^−1^
Trans-Fat (% of energy intake) ^c^			
Below the threshold of 1.5	7.35	0.79, 13.90	2.96 × 10^−2^
Above the threshold of 1.5	0.70	−1.89, 3.29	6.03 × 10^−1^
Dietary Cholesterol (mg/day) ^d^			
Below the threshold of 250	0.15	0.07, 0.23	5.98 × 10^−4^
Above the threshold of 250	0.02	−0.03, 0.07	3.61 × 10^−1^

^a^Multivariable model with omega-6, omega-3 (mg/day) and Gross National Income modeled with linear terms, and saturated fat, dietary cholesterol, and trans-fat were modeled with penalized splines (*n* = 183 countries).

^b^The same model as in a, but now with a linear term for saturated fat, and evaluated in two separate strata (above and below the threshold of 10% saturated fat by total energy intake).

^c^The same model as in a, but now with a linear term for trans-fat, and evaluated in two separate strata (above and below the threshold of 1.5% trans-fat by total energy intake).

^d^The same model as in a, but now with a linear term for dietary cholesterol, and evaluated in two separate strata (above and below the threshold of 250 mg/day of dietary cholesterol).

### Longitudinal analyses

The longitudinal within-country changes in lipid intakes between 1990 and 2010 were small (Table 5), but the unadjusted regression model still validated the association between omega-6 and ASAIR (Table 6; *β* = 0.0341; 95%CI: 0.0154, 0.0529; *p* = 4.70 × 10^−4^). Unadjusted regression models for the other lipids yielded findings that were generally consistent with the cross-sectional findings, but none of the associations reached significance (Table 6). The longitudinal relationship between omega-6 and ASAIR remained significant upon multivariable adjustment (Table 6; *β* = 0.0375; 95%CI: 0.0164, 0.0586; *p* = 6.50 × 10^−4^).

**Table 5:** Longitudinal changes in lipid intakes and ASAIR in the 183 countries

	Mean	Standard Deviation	Median	25th Percentile	75th Percentile	Min	Max
Outcome Variable							
Percent Change in ASAIR from 1990 to 2019^a^	0.012	0.111	0.020	-0.040	0.070	-0.420	0.580
Exposure Variables							
Percent Change in Omega6 from 1990 to 2010^b^	0.42	0.83	0.30	-0.10	0.70	-2.00	3.40
Percent Change in Omega-3-PUFA from 1990 to 2010 ^b^	58	79	41	9	94	-193	400
Percent Change in Saturated Fat from 1990 to 2010 ^b^	0.05	1.32	0.00	-0.60	0.50	-4.40	4.60
Percent Change in Trans-Fat from 1990 to 2010 ^b^	0.07	0.21	0.00	0.00	0.10	-0.80	1.50
Percent Change in Dietary Cholesterol from 1990 to 2010 ^b^	3.6	19.9	2.2	-10.3	15.7	-57.0	57.8
Change in Gross National Income from 1990 to 2010 ^c^	0.38	0.60	0	0	1	-1	2

^a^ASAIR estimates were obtained from Li et al., 2022^17^. They were produced by the *GBD Disease and Injury Incidence and Prevalence Collaborators* and downloaded from the IHME Global Health Data Exchange (http://ghdx.healthdata.org/gbd-results-tool) on March 20, 2020.

^b^Lipid estimates were obtained from Micha et al., 2015^14,15,18^. They were produced by the *Nutrition and Chronic Diseases Expert Group* (NutriCoDE) for the 2010 Global Burden of Diseases, Injuries, and Risk Factors Study, and they used a Bayesian hierarchical imputation model harmonize these estimates from diverse source data^14,15,18^.

^c^This variable was coded as the country development level change (based on per capita GNI) from 1990 to 2010. Development levels were not available for 27 countries in 1990, but we were able to obtain substitute country development baseline values for 24 of these countries by using an estimate from 1991 or 1992^26^. Thus, only Montenegro, Serbia, and Timor-Leste were missing country development level change data.

**Table 6:** Results from linear regression models of percent change in ASAIR from 1990 to 2019

	Percent Change in ASAIR from 1990 to 2019 associated with a 1% increase in country-level lipid intake (β)	95% CI	*p* value
Unadjusted Models			
Percent Change in Omega6 from 1990 to 2010	3.41 × 10^−02^	1.54 × 10^−02^, 5.29 × 10^−02^	4.70 × 10^−04^
Percent Change in Omega-3 from 1990 to 2010	9.63 × 10^−5^	−1.08 × 10^−4^, 3.01 × 10^−4^	3.58 × 10^−1^
Percent Change in Saturated Fat from 1990 to 2010			
Below the threshold of 10	4.38 × 10^−3^	−1.83 × 10^−2^, 2.70 × 10^−2^	7.06 × 10^−1^
Above the threshold of 10	−2.39 × 10^−3^	−1.71 × 10^−2^, 1.23 × 10^−2^	7.51 × 10^−1^
Percent Change in Trans-Fat from 1990 to 2010			
Below the threshold of 1.5	1.37 × 10^−1^	−2.43 × 10^−2^, 2.98 × 10^−1^	9.82 × 10^−2^
Above the threshold of 1.5	2.28 × 10^−2^	−6.33 × 10^−2^, 1.09 × 10^−1^	6.08 × 10^−1^
Percent Change in Dietary Cholesterolfrom 1990 to 2010			
Below the threshold of 250	9.23 × 10^−4^	−2.44 × 10^−4^, 2.09 × 10^−3^	1.24 × 10^−1^
Above the threshold of 250	2.14 × 10^−5^	−1.16 × 10^−3^, 1.20 × 10^-3^	9.72 × 10^−1^
Multivariable Adjusted Model^a^			
Percent Change in Omega-6 from 1990 to 2010	3.75 × 10^−02^	1.64 × 10^−02^, 5.86 × 10^−02^	6.50 × 10^−04^

^a^ Adjusted for: percent Change in omega-3, percent change in saturated fat, percent change in trans-fat, and percent change in dietary cholesterol, and change in Gross National Income level. To account for distinct associations on opposite sides of the threshold for saturated Fat, the saturated Fat variable was interacted with a dichotomous dummy variable for *change-occurred-above-or-below threshold*. Trans-Fat and dietary cholesterol also had thresholds and were modeled in the same way (*n* = 183 countries).

## Discussion

We observed a multivariable-adjusted positive linear association between mean country-level intake of omega-6 in 2010 and ASAIR in 2019. This association was then validated with a second multivariable adjusted model using longitudinal data. Specifically, we found that the percent change in the country-level omega-6 intake from 1990 to 2010 was positively associated with the percent change ASAIR between 1990 and 2019. These findings need further validation and trials to establish if the relationship is causal. However, the validation of this pattern in longitudinal data is among the strongest corroborations that can be made in non-experimental data. Well-designed prospective cohort studies with individual-level data would be best for individual level intake questions, but they are not ideal for this population level inquiry. With a sample size of 183 countries, we found strong associations in discovery and in longitudinal validation, and the *p*-values were exceptionally small for a study with this sample size. Our findings remain significant even under the most conservative adjustment for multiple testing (considering both discovery and confirmatory regressions as 18 separate tests the Bonferroni adjusted threshold for *α* = 0.05 would be *p* < 2.8 × 10^−3^) With respect to potential confounding bias we adjusted for all other measured lipids, per capita GNI (country income level), and the incidence rates were age standardized. Importantly, other systematic biases in mean lipid assessment and incidence rate determination were accounted for in the regression models that produced and harmonized the country level exposure and outcome data (lipids^14,15^, and ASAIR^17^). Additionally, we note that the longitudinal design controls for both measured and unmeasured potential confounders that are stable within the time course of the study. Finally, the risk of selection bias was reduced by including most of the countries on Earth. In short, generalizability is not a core concern because there is no clear need to make inferences beyond the participant countries.

Lipid epidemiology, particularly with respect to PUFAs, is a very complex area and a number of analytical problems have produced equivocal and sometimes contradictory findings for many outcomes^27,28^. While it is impossible to fully enumerate the issues, most of the individual level studies fail to account for the majority of the key factors that can alter measured effects^28,29^. These include: historical vs recent intake, potential nonlinearities (sufficiency thresholds), endogenous interconversion of ingested lipid precursors, biochemical interplay between omega-3 and omega-6, the error inherent in most individual level diet assessments, and potential confounding by other lipids^19,27–30^. Some of these issues have been addressed, but others remain ignored, or have been intractable in practice. One way to address several of these problems is to leverage biomarkers^11^. This can provide essential convergent evidence as it features distinct strengths and weaknesses^31,32^ when compared to studies of intake. However, biomarkers are not without issues, as the endogenous processing of lipids can lead to distinct levels between tissues, and the genetic background of the individual can greatly alter these mechanisms^33–35^. Therefore, lipid biomarker differences between individuals might reflect altered physiology, or altered intake, or both^36^.

As an example, de Oliveira Otto et al.^11^ evaluated circulating (blood) levels of omega-3 and omega-6 in relationship to cognitive function and dementia. They found protective associations with docosapentaenoic acid (C22:5n-3; an omega-3), but unlike much of the intake literature, they also observed protective associations with arachidonic acid (an omega-6). There could be a number of reasons why omega-6 findings differ between intake levels and blood levels, but metabolism and asymmetric tissue distributions may be involved. As an example, elevated Amyloid-β 42 (Aβ42) and Amyloid plaques in the brain is a hallmark of AD, but elevated Aβ42 in cerebrospinal fluid (CSF) is associated with decreased risk of AD^37^. Elevated brain levels may reflect pathological aggregation, and elevated CSF levels may indicate vigorous clearance. The authors astutely note that the patterns they observed could be due to metabolism and that blood levels of arachidonic acid appear to be under tight physiologic control (i.e. reflecting the determinants of individual physiology more than habitual or recent intake). Neuropathological assessments indicate that brain specific changes in PUFA metabolism may play a role in AD etiology^38^. Thus, processing and transport of these lipids need further attention, if we are to understand the differences between intake and circulating levels of PUFAs and their relationship to disease.

The association between omega-6 and AD that we report here is largely consistent with prior literature in individual level human studies and animal experiments^8,39–43^, but as we discussed, there are many technical hurdles in assessing this association, and not all studies are in agreement^11,44^. Further complicating this research area is the substitution of “deaths attributable to dementia”^45^ or AD prevalence, for AD incidence when attempting etiologic inference. When compared to incidence, “deaths attributable to dementia” and prevalence are much more likely to reflect non-etiologic factors (e.g., quality of medical care). While some studies have reported an inverse association between omega-3 and AD^8,46–48^, the results have been inconsistent^49,50^, and here we only observed a non-significant trend in this direction. Omega-3 and omega6 share an endogenous processing pathway^51,52^ involving fatty acid desaturases (*FADS*) and elongases of very long chain fatty acids (*ELOVL*). Interestingly, the gene clusters that produce these enzymes are both implicated in AD^39,53^. Thus, our findings are consistent with the hypothesis that the inflammatory endpoints of omega-6 processing are more important than the omega-3 physiology for increasing the risk of AD. While arachidonic acid is known to be a precursor in the production of many pro-inflammatory mediators (Fig. 1)^52^, and inflammation is known to be a key etiologic factor in AD etiology^54^, our findings cannot provide direct information on mechanisms. There is some evidence that the effects of arachidonic acid intake on cognitive decline may be ameliorated with antioxidants^55^. This does not clarify which if any omega-6 species are driving cognitive decline, but it indicates that the putative effect appears to depend on inflammation. These findings could be reconciled with the putative “protective effect” of circulating arachidonic acid^11^ if the conversion rate of the lipids in Fig. 1 (including arachidonic acid), is driving the cognitive effect. In other words, slower conversion rates may result in higher arachidonic acid levels and lower proinflammatory mediator levels. This hypothesis should be investigated.

The 2024 Lancet Report on Dementia^56^ raises critical issues with respect to AD comorbidities and competing risks that point to PUFA-imbalance driven inflammation. In short, the report notes that AD is linked to depression and multiple cardiovascular disease risk factors including hypertension and type2 diabetes. This area deserves further research because these and other conditions are frequently linked to PUFA imbalances^57–64^ and if this reflects a true causal mechanism, then they can serve as competing risks in AD development. Thus, omega-6 excess may cause a set of health problems that can partially mask its link to AD in epidemiologic analyses. This raises the hypothesis that addressing omega-6 excesses and omega-3 insufficiencies in food systems could yield multiplexed health benefits beyond AD.

The magnitude of the initial association with omega-6 (*β* = 2.44; 95%CI: 1.70, 3.19; *p* = 1.38 × 10^−9^) indicates that a one standard deviation decrease (−1.9% as a percent of total daily energy intake) could reduce ASAIR by 4.6 new cases of AD per 100,000 per year (2.44*1.9). For the US, which had an ASAIR of 110 new cases per 100,000 in 2019, a 2 standard deviation decrease in mean omega-6 intake would amount to 8.4% reduction in new cases annually ([110–9.2]/110 = 91.6%). We did not evaluate AD prevalence in this study because prevalence is affected by survival time, which can involve factors that are not relevant to disease etiology. However, if all other factors were held constant, lower annual incidence could compound over time to reduce the prevalence and societal burden of AD.

Our findings for saturated fat, trans-fat, and dietary cholesterol are also supported by the literature^2,65–68^, but the patterns were not significant in our longitudinal analyses. This is not surprising given the small magnitude of the longitudinal changes in lipid intakes that were observed within countries. Individual level studies, both cross-sectional and longitudinal, generally share this same limitation (i.e., small range of exposure). The results from our free-knot penalized spline regression models remain critical for future research as they indicate that nonlinear relationships should be considered in future inquiries of these lipids. If the relationships reported here are validated in subsequent work, then reductions in saturated fat, trans-fat, and dietary cholesterol may further reduce country-level ASAIR. For countries below the saturated fat threshold of 10% of total daily energy intake, a two standard deviation decrease in saturated fat intake would correspond to a reduction in ASAIR by 24.0 new cases of AD per 100,000 per year (2.67*9.0). Similar calculations for trans-fat (7.35*1.4) and dietary cholesterol (0.15*110) would predict 10.3 and 16.5 fewer new cases per 100,000 per year, respectively. In 2019, the US had a mean saturated fat intake of 11.8% of total daily energy intake, a mean trans-fat intake of 2.8% of total daily energy intake, and a mean dietary cholesterol of 296 mg/day. A two standard deviation decrease for each of these fats would lower the US mean intakes below the identified intake thresholds (10% of total daily energy intake for saturated fat, 1.5% of total daily energy intake for trans-fat, and 250 mg/day for dietary cholesterol). Such decreases would be predicted to reduce the number of new AD cases per 100,000 per year: 19.2 fewer due to saturated fat reductions (2.67*7.2), 0.7 fewer due to trans-fat reductions (7.35*0.1), and 9.6 fewer due to dietary cholesterol reductions (0.15*64). When added to the omega-6 findings, a two standard deviation decrease in omega-6, saturated fat, trans-fat, and dietary cholesterol could potentially yield a net reduction of 38.7 new cases per 100,000 per year in the US. This would amount to a 35.2% reduction in new cases of AD in the US each year. ([110−38.7]/110 = 64.8%). Again, the saturated fat, trans-fat, and dietary cholesterol findings still need to be corroborated on the country level, but there is substantial motivation to conduct follow-up studies because of the size of the putative benefits.

There are limitations to our work. First, we note that our country-level analyses depend on the accurate estimation of mean lipid intakes and ASAIR. These estimates come from peer reviewed publications and extensive care was made to account for potential biases. Given the thoroughness of the data harmonization methods, we do not expect large errors in exposure or outcome assessment, but we re-iterate that these are country-level patterns. We do not have information on within country racial and cultural subgroups that could contribute differently to any country’s mean intakes. Binning countries by presumed racial or cultural similarity would also not effectively address this issue. Second, we note that we had limited covariates available for confounding adjustment and this is a common issue in country-level analyses. However, we did adjust for all other lipids, and the incidence rates were age standardized. Additionally, we adjusted both our cross-sectional and longitudinal analyses for country development level as measured by GNI. This can reduce residual bias from factors that are correlated with country level economic improvement. We also emphasize that the longitudinal analyses intrinsically account for confounding factors that are stable in time. In other words, confounding by genetic and environmental factors that were stable within the time course of the analysis, was controlled for by study design. Of course, some unmeasured, and likely relevant factors, may not have been stable over the course of the study. Thus, we cannot rule out the possibility of unaddressed confounding that could happen if other causes of ASAIR (e.g., Air Pollution) were not stable over time and were strongly correlated with omega-6 intake changes. Perhaps most importantly, the omega-6 results were validated in the longitudinal models despite limited power (and the other lipid findings revealed supportive trends). Finally, we note that there were correlations between the lipid intakes and this could have created issues with collinearity. Despite the potential for collinearity, we observed multiple significant independent associations in these models between lipids and ASAIR when adjusting for all lipid intakes, indicating that collinearity was not a substantial concern.

A third limitation is that the units in the available data preclude the calculation of a standard omega-6/omega-3 ratio. Omega-3 is provided in mg/day but omega-6 is provided as a percent of total daily energy intake, and we lack the data to harmonize these units. While this is a limitation for comparison to prior work, in another way, it is a strength for developing better hypotheses. The relative amount of each class of PUFA can be important due to their shared biochemical pathway, but a ratio is still an imperfect method for dealing with this issue. The ratio approach prevents the consideration of potential sufficiency thresholds (nonlinearities) for each PUFA, and it models someone with 3 mg omega-6 and 1 mg omega-3 intake as having the exact same consumption as someone who eats 3 g omega-6 and 1 g omega-3. Additionally, by avoiding a ratio we are able to include the two PUFAs as independent terms in the same model. This avoids constraining the analysis by assuming a specific mathematical relationship between them^69^. We note that our study, like many prior studies, could not distinguish between the different species of omega-6. The patterns observed here and in prior studies could be driven by: 1) all omega-6, 2) subsets of omega-6, or 3) one specific omega-6 species (e.g. linoleic acid, arachidonic acid, or tetracosatetraenic acid). Future studies should seek to address this question, especially since biochemical pathways indicate that arachidonic acid and its precursors may be more important for producing mediators of inflammation than downstream omega-6 species (Fig. 1).

A fourth limitation is that the time lags do not perfectly align between the discovery and validation analyses. We would have preferred to have access to changes in ASAIR between 1999 and 2019 instead of 1990 to 2019. However, this is a minor point and the expected random error would likely bias toward the null. Finally, we note that while these findings are very useful for country-level inference and intervention, they are not directly applicable to individual level inference (the ecological fallacy). Therefore, although our results are compelling at the country level, the individual level will require additional study.

Overall, these findings may provide an approach for reducing ASAIR through food system interventions. If trials confirm our findings, then we can hone the prevention strategies that governments need as populations around the globe get older^70^. Importantly, this work stands on vast convergent evidence^32^ implicating lipid dysfunction in AD etiology^1–9^. It is now becoming clear that lipid abnormalities are causal factors^71^ in AD development, and though lipids have not always been the center of focus for AD researchers, the genetics have been consistently pointing in this direction for over 30 years^1,7^. Perhaps most importantly, if achieved, diet-based interventions could have large impact on AD incidence. While determining safe, optimal changes to country level lipid intakes needs some additional study, the benefits might extend beyond ASAIR, as excess omega-6 in our food systems is linked to many other serious and common chronic diseases^72,73^. The potential ASAIR benefits will vary by country, but based on our estimates for the US, a moderate decrease in omega-6 intake could reduce ASAIR by 8%. Additional decreases in saturated fat, trans-fat, and dietary cholesterol might yield total ASAIR reductions of 35%. Our findings are promising as they indicate a rather straightforward path to reducing AD burden.

## Supplementary information

Supplemnetary information

Description of Additional Supplementary files

Supplementary Data 1

Reporting summary

## Data Availability

The raw data are publicly available through the sources described in the methods and these links: https://www.bmj.com/content/348/bmj.g2272.long^14^; https://www.bmj.com/content/350/bmj.h1702^15^; 10.3389/fnagi.2022.937486^17^. Source data for the analyses, tables, figures, and Supplementary Figs. is in Supplementary Data 1.

## References

[1] Corder, E. H. et al. Gene dose of apolipoprotein E type 4 allele and the risk of Alzheimer’s disease in late onset families. *Science* **261**, 921–923 (1993). 8346443 10.1126/science.8346443

[2] Honda, T. et al. Serum elaidic acid concentration and risk of dementia: the Hisayama study. *Neurology* **93**, e2053–e2064 (2019). 31645469 10.1212/WNL.0000000000008464

[3] Abdullah, L. et al. APOE ε4 specific imbalance of arachidonic acid and docosahexaenoic acid in serum phospholipids identifies individuals with preclinical Mild Cognitive Impairment/Alzheimer’s Disease. *Aging* **9**, 964–985 (2017). 28333036 10.18632/aging.101203 PMC5391242

[4] Ebright, B. et al. Eicosanoid lipidome activation in post-mortem brain tissues of individuals with APOE4 and Alzheimer’s dementia. *Alzheimers Res. Ther.* **14**, 152 (2022). 36217192 10.1186/s13195-022-01084-7 PMC9552454

[5] Moulton M. J. et al. Neuronal ROS-induced glial lipid droplet formation is altered by loss of Alzheimer’s disease-associated genes. *Proc. Natl. Acad. Sci. USA*. 118. 10.1073/pnas.2112095118 (2021). 10.1073/pnas.2112095118 PMC8719885 34949639

[6] Thomas, M. H. et al. Dietary arachidonic acid increases deleterious effects of amyloid-β oligomers on learning abilities and expression of AMPA receptors: putative role of the ACSL4-cPLA(2) balance. *Alzheimers Res. Ther.* **9**, 69 (2017). 28851448 10.1186/s13195-017-0295-1 PMC5576249

[7] Kunkle, B. W. et al. Genetic meta-analysis of diagnosed Alzheimer’s disease identifies new risk loci and implicates Aβ, tau, immunity and lipid processing. *Nat. Genet.* **51**, 414–430 (2019). 30820047 10.1038/s41588-019-0358-2 PMC6463297

[8] Chew, H., Solomon, V. A. & Fonteh, A. N. Involvement of lipids in Alzheimer’s disease pathology and potential therapies. *Front. Physiol.* **11**, 598 (2020). 32581851 10.3389/fphys.2020.00598 PMC7296164

[9] Haney M. S. et al. APOE4/4 is linked to damaging lipid droplets in Alzheimer’s disease microglia. *Nature*. Published online March 13, 10.1038/s41586-024-07185-7 (2024). 10.1038/s41586-024-07185-7 PMC10990924 38480892

[10] Nwaru B. I. et al. Quality of dietary fat and risk of Alzheimer’s disease and dementia in adults aged ≥50 years: a systematic review. *Food Nutr. Res*. 66 10.29219/fnr.v66.8629 (2022). 10.29219/fnr.v66.8629 PMC9338447 35950105

[11] de Oliveira Otto, M. C. et al. Circulating omega-3 and omega-6 fatty acids, cognitive decline, and dementia in older adults. *J. Alzheimers Dis.* **95**, 965–979 (2023). 37638432 10.3233/JAD-230083 PMC10765383

[12] Rose, G. Sick individuals and sick populations. *Int. J. Epidemiol.* **14**, 32–38 (1985). 3872850 10.1093/ije/14.1.32

[13] Pearce, N. The ecological fallacy strikes back. *J. Epidemiol. Community Health* **54**, 326–327 (2000). 10814650 10.1136/jech.54.5.326 PMC1731667

[14] Micha, R. et al. Global, regional, and national consumption levels of dietary fats and oils in 1990 and 2010: a systematic analysis including 266 country-specific nutrition surveys. *BMJ* **348**, g2272 (2014). 24736206 10.1136/bmj.g2272 PMC3987052

[15] Global, regional, and national consumption levels of dietary fats and oils in 1990 and 2010: a systematic analysis including 266 country-specific nutrition surveys. *BMJ*. 350, h1702 10.1136/bmj.h1702 (2015). 10.1136/bmj.h1702 PMC4589816 25813336

[16] Wood S. N. Package’mgcv’: GAMs with GCV/AIC/REML smoothness estimation and GAMMs by PQL. November 30, 2010. Accessed November 30, 2010. https://cran.r-project.org/web/packages/mgcv/mgcv.pdf accessed 11/30/2010.

[17] Li, X. et al. Global, regional, and national burden of Alzheimer’s disease and other dementias, 1990-2019. *Front. Aging Neurosci.* **14**, 937486 (2022). 36299608 10.3389/fnagi.2022.937486 PMC9588915

[18] Micha, R. et al. Estimating the global and regional burden of suboptimal nutrition on chronic disease: methods and inputs to the analysis. *Eur. J. Clin. Nutr.* **66**, 119–129 (2012). 21915137 10.1038/ejcn.2011.147

[19] Ciesielski, T. H. & Williams, S. M. Low Omega-3 intake is associated with high rates of depression and preterm birth on the country level. *Sci. Rep.* **10**, 19749 (2020). 33184396 10.1038/s41598-020-76552-x PMC7661496

[20] Burdge, G. C., Jones, A. E. & Wootton, S. A. Eicosapentaenoic and docosapentaenoic acids are the principal products of alpha-linolenic acid metabolism in young men*. *Br. J. Nutr.* **88**, 355–363 (2002). 12323085 10.1079/BJN2002662

[21] Burdge, G. C. & Wootton, S. A. Conversion of alpha-linolenic acid to eicosapentaenoic, docosapentaenoic and docosahexaenoic acids in young women. *Br. J. Nutr.* **88**, 411–420 (2002). 12323090 10.1079/BJN2002689

[22] Calder, P. C. Docosahexaenoic acid. *Ann. Nutr. Metab.* **69**, 7–21 (2016). 27842299 10.1159/000448262

[23] Vos, T. et al. Global burden of 369 diseases and injuries in 204 countries and territories, 1990–2019: a systematic analysis for the Global Burden of Disease Study 2019. *Lancet* **396**, 1204–1222 (2020). 33069326 10.1016/S0140-6736(20)30925-9 PMC7567026

[24] Mork, D., Braun, D. & Zanobetti, A. Time-lagged relationships between a decade of air pollution exposure and first hospitalization with Alzheimer’s disease and related dementias. *Environ. Int.* **171**, 107694 (2023). 36521347 10.1016/j.envint.2022.107694 PMC9885762

[25] Blencowe, H. et al. National, regional, and worldwide estimates of preterm birth rates in the year 2010 with time trends since 1990 for selected countries: a systematic analysis and implications. *Lancet* **379**, 2162–2172 (2012). 22682464 10.1016/S0140-6736(12)60820-4

[26] Hamadehc N., Van Rompaey C., Metrea E. World Bank Group country classifications by income level. https://blogs.worldbank.org/opendata/new-world-bank-group-country-classifications-income-level-fy24 (Accessed March 12, 2024).

[27] Shim, J. S., Oh, K. & Kim, H. C. Dietary assessment methods in epidemiologic studies. *Epidemiol. Health* **36**, e2014009 (2014). 25078382 10.4178/epih/e2014009 PMC4154347

[28] Ciesielski T. H. Global access to uncontaminated Omega-3 polyunsaturated fatty acids requires attention. *AJPM Focus*. 10.1016/j.focus.2025.100341. 10.1016/j.focus.2025.100341 PMC12166692 40520676

[29] Ciesielski, T. H. n-3 and preterm birth: what can we learn from the heterogeneity? *Public Health Nutr.* **23**, 2453–2454 (2020). 32338238 10.1017/S1368980019004713 PMC11374574

[30] Ciesielski, T. H., Bartlett, J. & Williams, S. M. Omega-3 polyunsaturated fatty acid intake norms and preterm birth rate: a cross-sectional analysis of 184 countries. *BMJ Open* **9**, e027249 (2019). 10.1136/bmjopen-2018-027249 PMC6527982 31005937

[31] Ciesielski, T. H., Aldrich, M. C., Marsit, C. J., Hiatt, R. A. & Williams, S. M. Transdisciplinary approaches enhance the production of translational knowledge. *Transl. Res.* **182**, 123–134 (2017). 27893987 10.1016/j.trsl.2016.11.002 PMC5362296

[32] Ciesielski, T. H. et al. Diverse convergent evidence in the genetic analysis of complex disease: coordinating omic, informatic, and experimental evidence to better identify and validate risk factors. *BioData Min.* **7**, 10 (2014). 25071867 10.1186/1756-0381-7-10 PMC4112852

[33] Yang, L. G., March, Z. M., Stephenson, R. A. & Narayan, P. S. Apolipoprotein E in lipid metabolism and neurodegenerative disease. *Trends Endocrinol. Metab.* **34**, 430–445 (2023). 37357100 10.1016/j.tem.2023.05.002 PMC10365028

[34] Rebeck, G. W. The role of APOE on lipid homeostasis and inflammation in normal brains. *J. Lipid Res.* **58**, 1493–1499 (2017). 28258087 10.1194/jlr.R075408 PMC5538293

[35] Reynolds, L. M. et al. FADS genetic and metabolomic analyses identify the ∆5 desaturase (FADS1) step as a critical control point in the formation of biologically important lipids. *Sci. Rep.* **10**, 15873 (2020). 32985521 10.1038/s41598-020-71948-1 PMC7522985

[36] Mayeux, R. Biomarkers: potential uses and limitations. *NeuroRx J. Am. Soc. Exp. Neurother.* **1**, 182–188 (2004). 10.1602/neurorx.1.2.182 PMC534923 15717018

[37] Verbeek, SpiesP. E., van Groen, M. M. & Claassen, T. JAHR. Reviewing reasons for the decreased CSF Abeta42 concentration in Alzheimer disease. *Front. Biosci. Landmark Ed.* **17**, 2024–2034 (2012). 22652762 10.2741/4035

[38] Snowden, S. G. et al. Association between fatty acid metabolism in the brain and Alzheimer disease neuropathology and cognitive performance: a nontargeted metabolomic study. *PLOS Med.* **14**, e1002266 (2017). 28323825 10.1371/journal.pmed.1002266 PMC5360226

[39] Hammouda, S. et al. Genetic variants in FADS1 and ELOVL2 increase level of arachidonic acid and the risk of Alzheimer’s disease in the Tunisian population. *Prostaglandins Leukot. Ess. Fat. Acids* **160**, 102159 (2020). 10.1016/j.plefa.2020.102159 32682282

[40] Sanchez-Mejia, R. O. & Mucke, L. Phospholipase A2 and arachidonic acid in Alzheimer’s disease. *Biochim. Biophys. Acta* **1801**, 784–790 (2010). 20553961 10.1016/j.bbalip.2010.05.013 PMC3024142

[41] Sanchez-Mejia, R. O. et al. Phospholipase A2 reduction ameliorates cognitive deficits in a mouse model of Alzheimer’s disease. *Nat. Neurosci.* **11**, 1311–1318 (2008). 18931664 10.1038/nn.2213 PMC2597064

[42] Thomas, M. H., Pelleieux, S., Vitale, N. & Olivier, J. L. Dietary arachidonic acid as a risk factor for age-associated neurodegenerative diseases: Potential mechanisms. *Biochimie* **130**, 168–177 (2016). 27473185 10.1016/j.biochi.2016.07.013

[43] Goozee, K. et al. Alterations in erythrocyte fatty acid composition in preclinical Alzheimer’s disease. *Sci. Rep.* **7**, 676 (2017). 28386119 10.1038/s41598-017-00751-2 PMC5429676

[44] Heude, B., Ducimetière, P. & Berr, C. Cognitive decline and fatty acid composition of erythrocyte membranes–the EVA study. *Am. J. Clin. Nutr.* **77**, 803–808 (2003). 12663275 10.1093/ajcn/77.4.803

[45] Wu, J. H. Y. et al. Circulating omega-6 polyunsaturated fatty acids and total and cause-specific mortality: the Cardiovascular Health Study. *Circulation* **130**, 1245–1253 (2014). 25124495 10.1161/CIRCULATIONAHA.114.011590 PMC4189990

[46] Hosseini, M., Poljak, A., Braidy, N., Crawford, J. & Sachdev, P. Blood fatty acids in Alzheimer’s disease and mild cognitive impairment: a meta-analysis and systematic review. *Ageing Res Rev.* **60**, 101043 (2020). 32194194 10.1016/j.arr.2020.101043

[47] Zhu, R. Z., Chen, M. Q., Zhang, Z. W., Wu, T. Y. & Zhao, W. H. Dietary fatty acids and risk for Alzheimer’s disease, dementia, and mild cognitive impairment: A prospective cohort meta-analysis. *Nutr. Burbank Los Angel Cty Calif.* **90**, 111355 (2021). 10.1016/j.nut.2021.111355 34218119

[48] Gustafson, D. R. et al. Dietary fatty acids and risk of Alzheimer’s disease and related dementias: observations from the Washington Heights-Hamilton Heights-Inwood Columbia Aging Project (WHICAP). *Alzheimers Dement J. Alzheimers Assoc.* **16**, 1638–1649 (2020). 10.1002/alz.12154 PMC8409226 32715635

[49] Alsumari, S. R., AlNouri, D. M., El-Sayed, M. M. A., El-Din, M. F. S. & Arzoo, S. The sociodemographic characteristics and dietary and blood plasma fatty acid profiles of elderly Saudi women with Alzheimer disease. *Lipids Health Dis.* **18**, 77 (2019). 30927917 10.1186/s12944-019-1029-0 PMC6441169

[50] Devore, E. E. et al. Dietary intake of fish and omega-3 fatty acids in relation to long-term dementia risk. *Am. J. Clin. Nutr.* **90**, 170–176 (2009). 19474131 10.3945/ajcn.2008.27037 PMC2696999

[51] Zhang, J. Y., Kothapalli, K. S. & Brenna, J. T. Desaturase and elongase-limiting endogenous long-chain polyunsaturated fatty acid biosynthesis. *Curr. Opin. Clin. Nutr. Metab. Care* **19**, 103–110 (2016). 26828581 10.1097/MCO.0000000000000254 PMC4768719

[52] Balić, A., Vlašić, D., Žužul, K., Marinović, B. & Bukvić Mokos, Z. Omega-3 versus omega-6 polyunsaturated fatty acids in the prevention and treatment of inflammatory skin diseases. *Int. J. Mol. Sci.* **21**, 741 (2020). 31979308 10.3390/ijms21030741 PMC7037798

[53] Peña-Bautista C., Álvarez-Sánchez L., Cañada-Martínez A. J., Baquero M., Cháfer-Pericás C. Epigenomics and lipidomics integration in Alzheimer disease: pathways involved in early stages. *Biomedicines*. 9, 1812 10.3390/biomedicines9121812 (2021). 10.3390/biomedicines9121812 PMC8698767 34944628

[54] Akiyama, H. et al. Inflammation and Alzheimer’s disease. *Neurobiol. Aging* **21**, 383–421 (2000). 10858586 10.1016/s0197-4580(00)00124-x PMC3887148

[55] Assmann, K. E., Adjibade, M., Hercberg, S., Galan, P. & Kesse-Guyot, E. Unsaturated fatty acid intakes during midlife are positively associated with later cognitive function in older adults with modulating effects of antioxidant supplementation. *J. Nutr.* **148**, 1938–1945 (2018). 30517725 10.1093/jn/nxy206

[56] Livingston, G. et al. Dementia prevention, intervention, and care: 2024 report of the Lancet standing Commission. *Lancet* **404**, 572–628 (2024). 39096926 10.1016/S0140-6736(24)01296-0

[57] Ciesielski T. Our limited and declining global access to uncontaminated omega3 polyunsaturated fatty acids requires attention. Poster Presentation presented at: ISEE 2024: 36th Annual Conference of the International Society of Environmental Epidemiology; August 26, Santiago, Chile. https://ehp.niehs.nih.gov/doi/abs/10.1289/isee.2024.1596 (2024).

[58] Middleton P. et al., Omega-3 fatty acid addition during pregnancy. *Cochrane Database Syst Rev*. Cd003402. 10.1002/14651858.CD003402.pub3 (2018). 10.1002/14651858.CD003402.pub3 PMC6516961 30480773

[59] Lu, Y., Qiao, D. & Mi, G. Clinical impacts of n-3 fatty acids supplementation on depression symptoms: an umbrella review of meta-analyses. *Br. J. Nutr.* **131**, 841–850 (2024). 37886879 10.1017/S000711452300226X

[60] Abdelhamid, A. S. et al. Omega-3 fatty acids for the primary and secondary prevention of cardiovascular disease. *Cochrane Database Syst. Rev.* **3**, CD003177 (2020). 32114706 10.1002/14651858.CD003177.pub5 PMC7049091

[61] Chao, T., Sun, J., Ge, Y. & Wang, C. Effect of omega-3 fatty acids supplementation on the prognosis of coronary artery disease: A meta-analysis of randomized controlled trials. *Nutr. Metab. Cardiovasc. Dis. NMCD* **34**, 537–547 (2024). 38161115 10.1016/j.numecd.2023.10.035

[62] Akbar, S., Rahman, A., Ahmad, N., Imran, M. & Hafeez, Z. Understanding the role of polyunsaturated fatty acids in the development and prevention of cancer. *Cancer Treat. Res* **191**, 57–93 (2024). 39133404 10.1007/978-3-031-55622-7_3

[63] Yan, J. H., Guan, B. J., Gao, H. Y. & Peng, X. E. Omega-3 polyunsaturated fatty acid supplementation and non-alcoholic fatty liver disease: A meta-analysis of randomized controlled trials. *Medicine* **97**, e12271 (2018). 30212963 10.1097/MD.0000000000012271 PMC6155966

[64] Zhang, Y. et al. Higher ratio of plasma omega-6/omega-3 fatty acids is associated with greater risk of all-cause, cancer, and cardiovascular mortality: A population-based cohort study in UK Biobank. *eLife* **12**, RP90132 (2024). 38578269 10.7554/eLife.90132 PMC10997328

[65] Kalmijn, S. et al. Dietary fat intake and the risk of incident dementia in the Rotterdam Study. *Ann. Neurol.* **42**, 776–782 (1997). 9392577 10.1002/ana.410420514

[66] Grant, W. B. & Blake, S. M. Diet’s role in modifying risk of Alzheimer’s disease: history and present understanding. *J. Alzheimers Dis. JAD* **96**, 1353–1382 (2023). 37955087 10.3233/JAD-230418 PMC10741367

[67] Solomon, A., Kivipelto, M., Wolozin, B., Zhou, J. & Whitmer, R. A. Midlife serum cholesterol and increased risk of Alzheimer’s and vascular dementia three decades later. *Dement. Geriatr. Cogn. Disord.* **28**, 75–80 (2009). 19648749 10.1159/000231980 PMC2814023

[68] Morris, M. C., Evans, D. A., Bienias, J. L., Tangney, C. C. & Wilson, R. S. Dietary fat intake and 6-year cognitive change in an older biracial community population. *Neurology* **62**, 1573–1579 (2004). 15136684 10.1212/01.wnl.0000123250.82849.b6

[69] Barr, D. B. et al. Urinary creatinine concentrations in the U.S. population: implications for urinary biologic monitoring measurements. *Environ. Health Perspect.* **113**, 192–200 (2005). 15687057 10.1289/ehp.7337 PMC1277864

[70] Feigin, V. L. et al. The global burden of neurological disorders: translating evidence into policy. *Lancet Neurol.* **19**, 255–265 (2020). 31813850 10.1016/S1474-4422(19)30411-9 PMC9945815

[71] Rothman, K. J. Causes. *Am. J. Epidemiol.* **104**, 587–592 (1976). 998606 10.1093/oxfordjournals.aje.a112335

[72] Patterson, E., Wall, R., Fitzgerald, G. F., Ross, R. P. & Stanton, C. Health implications of high dietary omega-6 polyunsaturated Fatty acids. *J. Nutr. Metab.* **2012**, 539426 (2012). 22570770 10.1155/2012/539426 PMC3335257

[73] Mariamenatu, A. H. & Abdu, E. M. Overconsumption of omega-6 polyunsaturated fatty acids (PUFAs) versus deficiency of omega-3 PUFAs in modern-day diets: the disturbing factor for their “balanced antagonistic metabolic functions” in the human body. *J. Lipids* **2021**, 8848161 (2021). 33815845 10.1155/2021/8848161 PMC7990530

