# Supplementary material for: Country-level incidence of Alzheimer disease and related dementias is associated with increased omega-6-PUFA consumption
Source: Commun Med (Lond). 2025 Jul 31;5:326. doi: 10.1038/s43856-025-01059-3 (PMC12314086; doi:10.1038/s43856-025-01059-3)

**Supplementary Information**

**Country Level Incidence of Alzheimer Disease and Related Dementias is Associated with Increased Omega-6-PUFA Consumption**

Timothy H. Ciesielski^*a^, Giuseppe Tosto^b^, Razaq O. Durodoye^a^, Farid Rajabali^c^, Rufus O. Akinyemi^d^, Goldie S. Byrd^e^, William S. Bush^a,f^, Brian W. Kunkle^c^, Christiane Reitz, Jeffery M. Vance^c^, Margaret A. Pericak-Vance^d^, Jonathan L. Haines^a,f^, and Scott M. Williams^a,f^

a Department of Population and Quantitative Health Sciences, Case Western Reserve University School of Medicine, 10900 Euclid Ave. Cleveland, OH 44106

b Taub Institute for Research on Alzheimer Disease and the Aging Brain,

Department of Neurology, Columbia University College of Physicians and Surgeons, 630 West 168th Street New York, NY 10032

c John P. Hussman Institute for Human Genomics, University of Miami Miller School of Medicine 1501 NW 10th Ave. Biomedical Research Building, Miami, FL 33136

d Neuroscience and Ageing Research Unit, Institute for Advanced Medical Research and Training, College of Medicine, University of Ibadan, P.M.B 5017 G.P.O Ibadan,Oyo State, Nigeria.

e Maya Angelou Center for Health Equity, Wake Forest University School of Medicine, 525 Vine Street Suite #150, 1st Floor, Winston-Salem, NC 27101

f Cleveland Institute for Computational Biology, Case Western Reserve University, 2-530 Wolstein Research Building, 2103 Cornell Road, Cleveland, OH

^*^ **Corresponding Author:**

Timothy H. Ciesielski Sc.D. M.D. M.P.H

Research Scientist, Department of Population and Quantitative Health Sciences

Case Western Reserve University School of Medicine

2210 Circle Drive, Robbins Building, Office E260, Cleveland, Ohio 44106

**Supplementary Figure 1:**

**Scatterplot of ASAIR by Country Level Omega-6 Intake**

Each dot represents one country in the analysis. Darker shading occurs when two dots overlap.


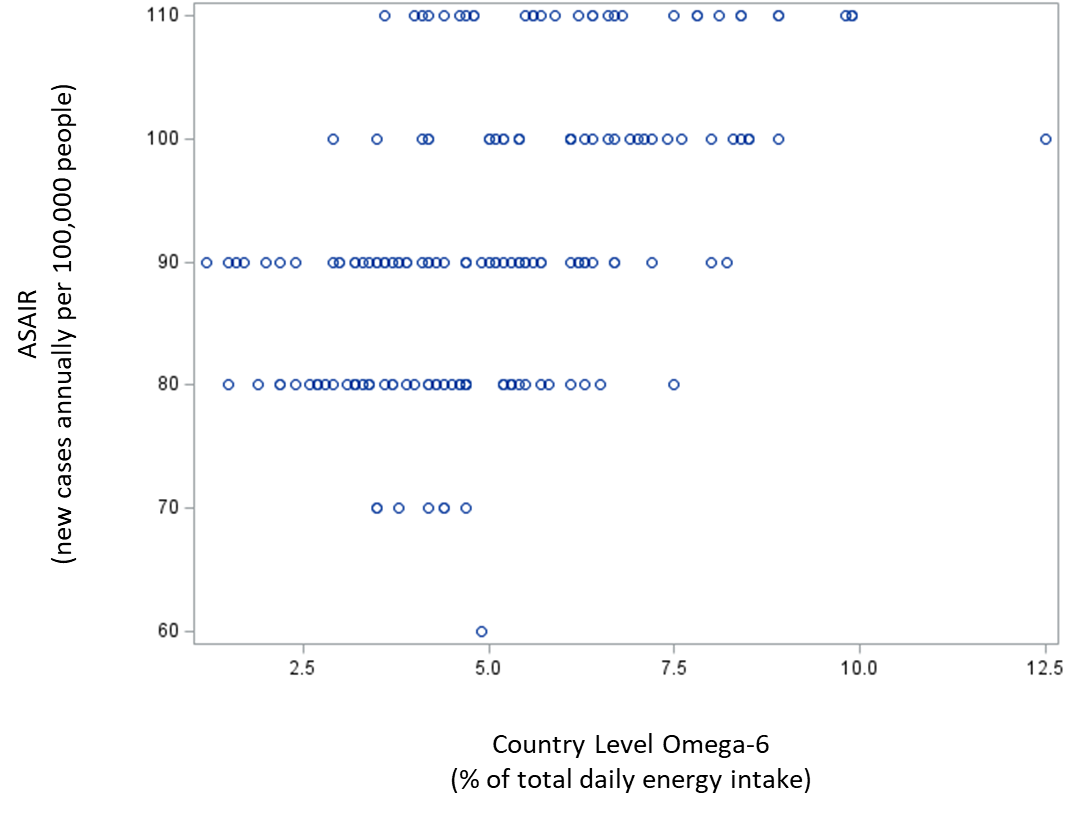


**Supplementary Figure 2:**

**Scatterplot of ASAIR by Country Level Omega-3 Intake**

Each dot represents one country in the analysis. Darker shading occurs when two dots overlap.


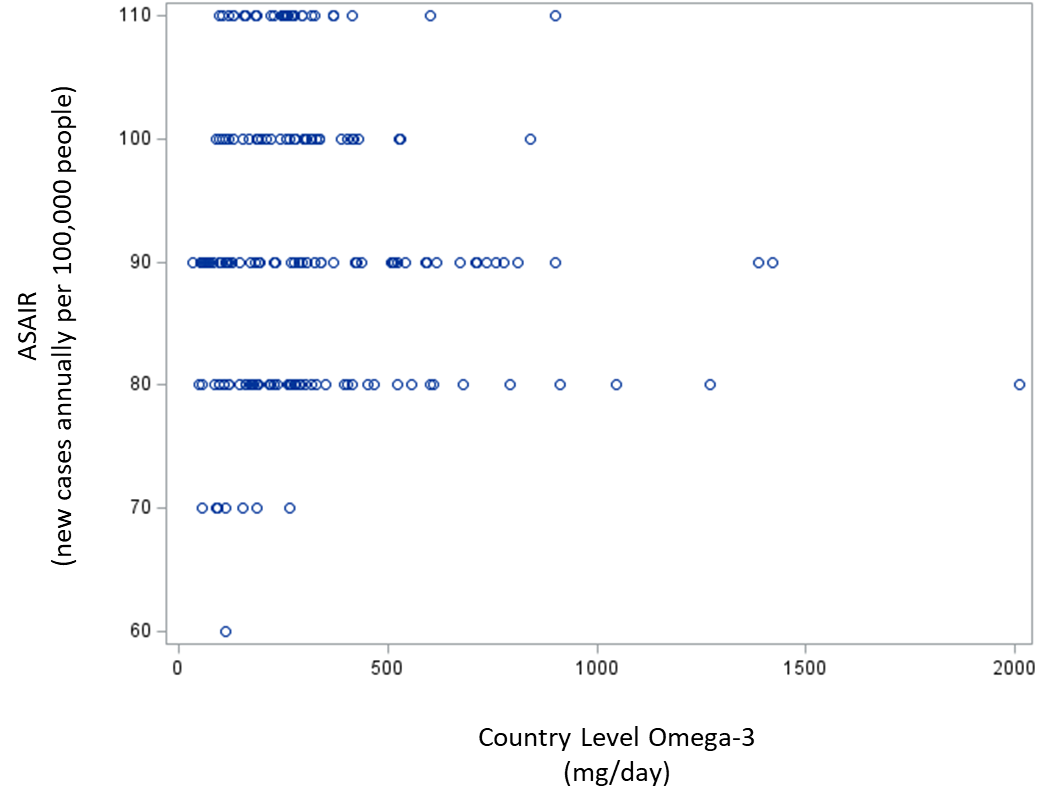


**Supplementary Figure 3:**

**Scatterplot of ASAIR by Country Level Trans Fat Intake**

Each dot represents one country in the analysis. Darker shading occurs when two dots overlap.


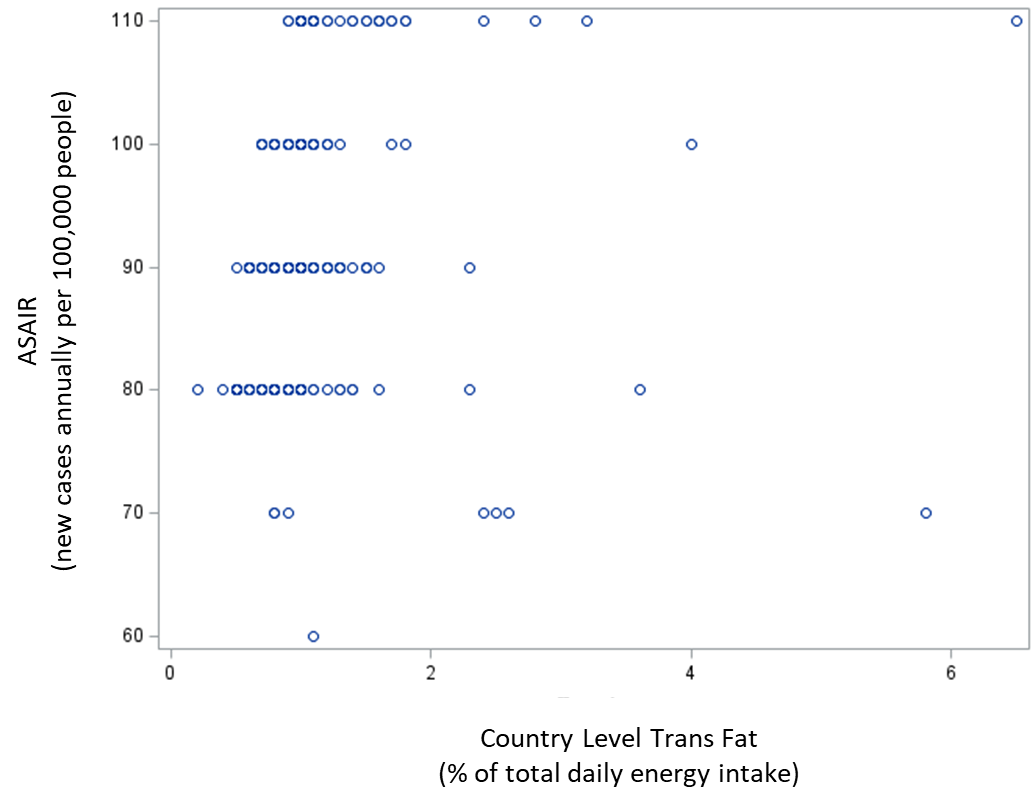


**Supplementary Figure 4:**

**Scatterplot of ASAIR by Country Level Saturated Fat Intake**

Each dot represents one country in the analysis. Darker shading occurs when two dots overlap.


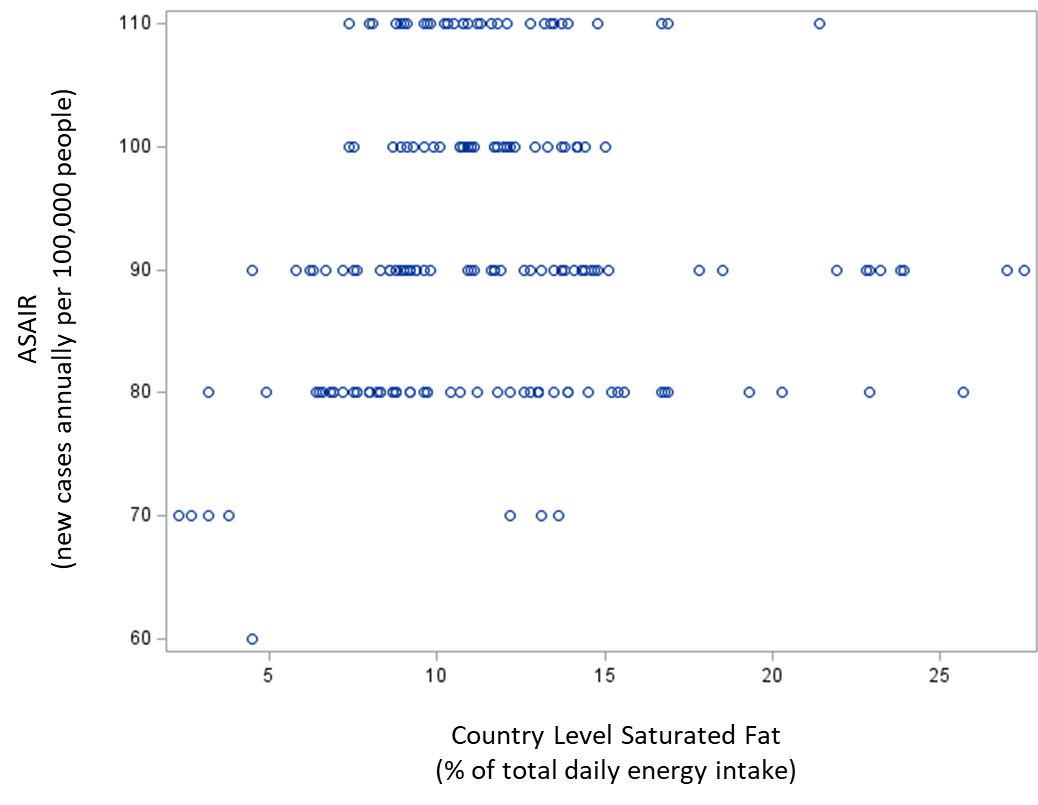


**Supplementary Figure 5:**

**Scatterplot of ASAIR by Country Level Cholesterol Intake**

Each dot represents one country in the analysis. Darker shading occurs when two dots overlap.


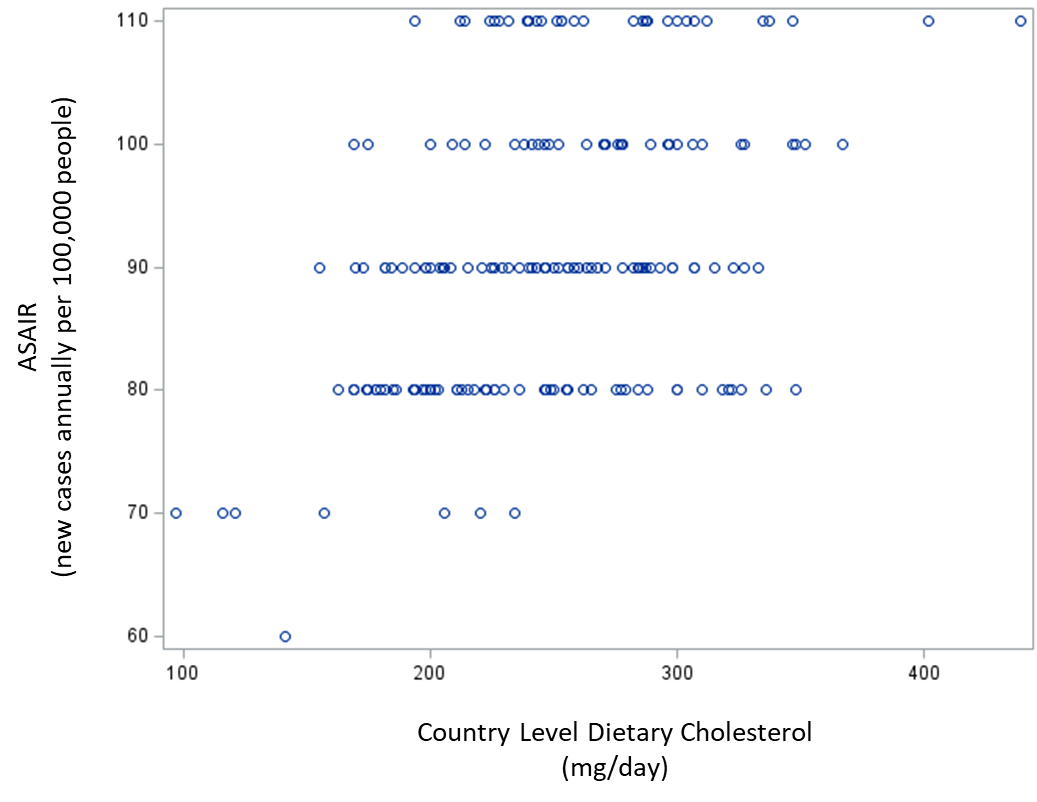

Supplement: Supplementary file 1 — Supplemnetary information [file 43856_2025_1059_MOESM1_ESM.docx]
