# Supplementary material for: Country-level incidence of Alzheimer disease and related dementias is associated with increased omega-6-PUFA consumption
Source: Commun Med (Lond). 2025 Jul 31;5:326. doi: 10.1038/s43856-025-01059-3 (PMC12314086; doi:10.1038/s43856-025-01059-3)
Supplement: Supplementary file 2 — Description of Additional Supplementary files [file 43856_2025_1059_MOESM2_ESM.pdf]

Description of Additional Supplementary files

File name: Supplementary Data 1

Description: The source data that supports the manuscript and Supplementary Figure 1.
